# Procrastination Mediates the Relationship between Problematic TikTok Use and Depression among Young Adults

**DOI:** 10.3390/jcm13051247

**Published:** 2024-02-22

**Authors:** Aleksandra M. Rogowska, Aleksandra Cincio

**Affiliations:** Institute of Psychology, University of Opole, 45-052 Opole, Poland

**Keywords:** depression, emerging adulthood, gender differences, Poland, problematic TikTok use, procrastination

## Abstract

**Background:** Although the prevalence of depression has increased significantly in recent years, especially in the young adult population, little is known about its causes and risk factors. The study aims to examine the mediating role of TikTok use in the relationships between procrastination and depression in young adults. **Methods:** A sample of 448 adults, ranging in age from 18 to 35 years (M = 24.45, SD = 3.76), including 214 men (48%), participated in the study. The cross-sectional survey consists of a modified Bergen Facebook Addiction Scale (BFAS) to assess problematic TikTok use (PTTU), the Pure Procrastination Scale (PPS) for procrastination measurement, and the nine-item Patient Health Questionnaire (PHQ-9) for screening depression symptoms. **Results:** The independent samples *t*-test indicates that emerging adults (ages ranging between 18 and 25 years) have more severe depression symptoms than young adults (26–35 years old). Gender differences were not found for procrastination, PTTU, and depression symptoms. Positive correlations were found between procrastination, PTTU, and depression symptoms. PTTU plays a mediating role in the associations between procrastination and depression. **Conclusions:** Both procrastination and PTTU treatment should be prioritized in the prevention and intervention programs for improving mental health among young adults. Some effective therapeutic methods are recommended.

## 1. Introduction

Recent research suggests that social media and smartphone overuse play a crucial role in daily lives, increasing depression and anxiety in young adults [1]. Depression is one of the most common, chronic, and costly disabilities affecting adults worldwide [2]. The World Health Organization (WHO) [2] reported in 2023 that around 3.8% of the world’s population suffers from depression, encompassing 5% of adults (including 4% among men and 6% among women). Globally, an estimated 280 million individuals are affected by depression, with it being approximately 50% more prevalent among women than men. The burden of depression on the population was deepened by the COVID-19 pandemic [3]. Among emerging adults, defined as individuals between 18 and 25 years of age, 25% experience depression [4]. This is the highest incidence and cumulative prevalence of depression of any age group, which is determined by the transition from late adolescence to young adulthood [5]. Problematic social media use can be understood as a non-substance-related disorder characterized by an excessive preoccupation and compulsion to engage with social media platforms, even in the face of adverse consequences [6]. Social media addiction, as one of potential dimensions of behavioral addiction, is characterized by being overly concerned about social media, driven by an uncontrollable urge to use it, and devoting so much time and effort to it that it impairs other important areas of life, often leading to adverse outcomes such as psychological distress, impaired real-life relationships, academic or occupational difficulties, decreased levels of physical activity and self-esteem, and heightened levels of loneliness, anxiety, depression, and poor quality of life and overall well-being [6,7,8,9,10,11,12]. Individuals experiencing problematic social media use may find it challenging to control the amount of time spent on social media platforms, leading to neglect of other important aspects of their lives [13].

Problematic social media use can involve various categories of digital platforms that facilitate online interaction, communication, and content sharing [14,15]. The social media landscape is constantly evolving, particularly among teenagers, who tend to be at the forefront of these changes [16]. According to a recent survey by the Pew Research Center [17] focusing on American teenagers aged 13 to 17, TikTok has experienced a significant surge in popularity since its introduction to North America several years ago. Approximately 67% of teens report using TikTok at some point, with 16% stating that they use it almost constantly. In contrast, the usage of Facebook, which was a dominant social media platform among teens in the Center’s 2014–2015 survey, has sharply declined from 71% at that time to 32% today [17]. The TikTok app, accessible on both Android and Apple smartphones, enables the creation, viewing, and sharing of videos and viral challenges, resulting in receiving likes, comments, and followers [18,19,20]. TikTok possesses one of the most sophisticated algorithmic systems, making it more captivating than other social media platforms in terms of addiction [21,22]. This behavior triggers the dopaminergic reward system that forms the foundation of addictive behaviors [23,24,25]. Research also indicates that mental health challenges stemming from the use of social media disproportionately impact women compared to men and vary depending on the specific social media platform and patterns of its use [26,27]. Statistics suggest [28] that among TikTok customers aged 18 to 24, women prevailed over men (24% vs. 18% of global users, respectively). Among TikTok’s audience, 31% are between 25 and 34 years old. Additionally, TikTok is more often indicated as the favorite social media platform among women (11.3%) than among men (6.6%). 

Psychological or behavioral dependence on social media platforms can lead to significant adverse impacts on individuals’ daily functioning. In particular, excessive use of social media during the COVID-19 pandemic increases the number of adverse consequences in children and adolescents, including behavioral problems, online grooming, addiction, insomnia, stress, anxiety and depression symptoms, headache, physical activity, body image and eating disorders, and sex-related issues [29,30]. Previous studies highlight various detrimental effects of social media on the mental health and overall well-being of people regardless of age [6,13,31,32,33,34,35]. Review studies and meta-analyses showed correlations between problematic social media use and heightened symptoms of depression, anxiety, stress, and decreased well-being [6,12,16,34,35,36,37,38,39,40,41,42,43], especially among adolescents and young adults. 

Excessive use of social media is associated with postponing important matters, which increases stress and worsens mood and general well-being. Procrastination is defined as the voluntary delay of an intended action despite the recognition that this delay may have adverse effects, such as compromising performance or increasing stress levels unnecessarily [44,45,46]. Procrastination occurs despite an individual having the capability to complete tasks, and it is typically accompanied by a subjective feeling of discomfort, stress, and anxiety [47,48]. Procrastination can manifest in various aspects of life, such as work, academic responsibilities, personal goals, or daily chores [49,50]. Procrastination is consistent over time. Therefore, it can be considered a personality trait, in particular as the most central facet of conscientiousness [46]. A meta-analysis [46] suggested that procrastination is a common behavior but that its prevalence can vary among individuals and across different contexts. Studies found that an estimated 20% of adults around the world (including Poland) are chronic procrastinators, namely individuals who intentionally delay self-sabotaging strategy decisions and tasks that need to be carried out [45,51,52]. Studies suggest that procrastination may be more prevalent among adolescents and emerging adults (especially among men) compared to older groups [47,51,53]. Gender differences were not found in procrastination in people over 30 years of age [47,52]. 

Procrastination is considered a self-regulatory failure, where individuals struggle to manage their time effectively and may prioritize short-term pleasure or avoidance of discomfort over long-term goals and responsibilities [44,46]. Unfortunately, these strategies can lead to increased stress, reduced productivity, decreased self-esteem, and a sense of guilt or frustration [54]. Another negative consequence of mental health is increased levels of anxiety and depression symptoms [46,53,54,55]. Higher levels of academic procrastination were positively related to depression and suicide ideation among college students during the COVID-19 pandemic [56]. Procrastination is related to social media use, as suggested in a recent study [21,49,57,58,59,60,61]. There is also some evidence suggesting that TikTok can be associated with procrastination, as the app’s addictive nature can lead users to engage in unhealthy habits and avoid tasks that require focus and effort [59].

### The Current Study Aims

The current study aims to explain the effect of the interplay between TikTok use and procrastination on depression in young adults. Given the widespread use of social media, understanding its impact on mental health is not only relevant at an individual level but also at a public health level. Addressing social media addiction and its connection to depression can have broader implications for public well-being. Emerging adults have the highest prevalence of depression symptoms compared to any other age population. Explanations of this phenomenon should be prioritized in social sciences and clinical studies. Social media platforms have become integral parts of people’s lives globally during the last two decades. The civilizing change in lifestyle relating to excessive use of social media can affect young people (the most vulnerable group) to a greater degree, causing a decrease in their well-being. Research indicates an association between the problematic use of social media and depression. However, the structure and preferences of particular social media use change continuously. The ever-changing nature of social media poses a challenge for researchers in determining whether problematic social media use should be recognized as a distinct clinical condition or as a manifestation of underlying psychiatric disorders. Therefore, diagnosing depression and the potential risk factors is important for preparing appropriate prevention and intervention programs. 

Although various social media platforms were examined previously, the association between depression and the relatively new TikTok platform, with dynamically growing popularity (especially among young people), was not exhaustively studied. In particular, the role of TikTok use in developing depression is unknown. Similarly, although a link between procrastination and depression was previously found, the mechanism of this association is unknown. An understanding of the relationship between procrastination, TikTok use, and depression is crucial due to the widespread adoption of the TikTok platform. The present research will examine the mediating role of problematic TikTok use on the relationship between procrastination and depression among emerging adults. We expect that chronic procrastinators will use TikTok as a coping strategy to reduce stress and tension related to unfinished, important matters. This strategy, however, increases stress and depression symptoms if it lasts too long and the TikTok use is getting out of control (problematic TikTok use). Procrastination (considered a personality trait) is a primary factor affecting both problematic TikTok use (behavioral addiction) and depression (psychological disorder). Investigating the association between procrastination, problematic TikTok use, and depression is essential for promoting mental health awareness, guiding intervention strategies, and fostering a more balanced and positive use of social media in society.

Procrastination will be treated in this study as a personality factor that increases Problematic TikTok use (PTTU) within the Interaction of Person-Affect-Cognition-Execution (I-PACE) model [62]. The I-PACE model offers a theoretical structure that differentiates between predisposing factors and variables that moderate or mediate their interactions with mental health [62]. Consistent with the I-PACE model, we assume that PTTU mediates the relationship between the predisposition to procrastination (understood as a personality trait) and depression (considered as a negative consequence of stabilization and intensification of excessive TikTok use). The shift from gratification to compensation (as the main motive of TikTok use) can explain the addiction process. PTTU is associated with spending time on entertainment instead of pursuing important goals, which increases stress and anxiety (because essential goals are not achieved) and ultimately increases depression. People with chronic procrastination, characterized by low self-control levels and a greater tendency to avoid unpleasant activities and duties, can spend more time using social media (including TikTok), which, in turn, could lead to a shift in the precious resource of time and increase procrastination [63,64,65,66]. 

Indeed, previous research seems to confirm our assumptions. Research suggests that procrastination correlated positively and moderately with depression and problematic smartphone use (PSU), while depression was weakly and positively related to PSU [67]. Previous research indicates that procrastination has a direct effect on well-being and depression [54]. Furthermore, the use of various social media has a significant direct impact on increasing depression [16,40,68,69]. Excessive use of cell phones increases depression, anxiety, and self-regulation, as suggested by longitudinal studies [69,70]. Also, recent studies showed that problematic TikTok use (PTTU) is a significant predictor of depression [71,72,73,74,75,76]. Finally, Davis et al. [8] found positive moderate correlations between all four dimensions of the newly developed Online Cognition Scale (OCS; loneliness and depression, diminished impulse control, distraction, and social comfort) as a measure of problematic internet use and both procrastination and depressive symptoms among undergraduates. However, the mediating effect of TikTok use on the relationship between procrastination and depression will be examined in this study for the first time. Moreover, the gender and age differences in procrastination, TikTok use, and depression will be taken into account in this study. Consistent with previous studies, we hypothesize:

**H1.**  *Women scored higher than men in PTTU [28] and depression symptoms [2], but men present higher levels of procrastination than women [47,53]. *

**H2.**  *Emerging adults (aged 18–25) scored higher than the young adult sample (aged 26–35) in procrastination [47], PTTU [17,28], and depression [4].*

**H3.**  *Procrastination correlates positively with PTTU [21,49,57,58,59,60,61,67] and depression symptoms [46,54,55,67,77], and PTTU is positively associated with depression symptom severity [67,71,72,73,74,75,76,78].*

**H4.**  *Consistent with the I-PACE model [62], we assume that PTTU mediates the relationship between the predisposition to procrastination (understood as a personality trait) and depression.*

## 2. Materials and Methods

### 2.1. Study Design and Procedure

The online cross-sectional survey, conducted using Google Forms, involved distributing a link and instructions on Facebook from September to January 2022. This information was shared within different groups dedicated to collaborative survey engagement among students and doctoral candidates. Those who willingly participated in the survey were given details about the study and instructions. Subsequently, upon giving informed consent for their participation, individuals completed a standardized set of questionnaires anonymously. Inclusion criteria included being over 18 years of age and using the TikTok app at least once in their life. Statistics suggest that TikTok is predominantly used among teenagers and young adults, and 83.4% of TikTok customers also use Facebook [28]. Furthermore, Facebook is the most-used global social media platform in terms of the number of users, whereas TikTok currently ranks sixth among all social media [28]. Taking into account the required age of participants (minimum 18 years) and the availability of relatively young age groups, we decided to use a conventional study group using Facebook because all students use this medium to communicate on matters related to their studies. Initially, 476 people answered the invitation, but 26 respondents were excluded because they never used TikTok, and two were younger than 18 years old. 

### 2.2. Participants’ Characteristic

A total of 448 individuals participated in the research, comprising 214 men (Table 1). The participants’ ages ranged from 18 to 35 years (*M* = 24.45, *SD* = 3.76). Among the participants, the majority indicated having secondary education (52%) and being students (52%), while 55% reported being in a relationship. Regarding the frequency of TikTok usage, the highest percentage of respondents (35%) used TikTok up to five times a day, typically spending up to one hour on the platform (38%). The primary motivation for using TikTok among participants was entertainment, cited by 33% of respondents. When it comes to how individuals spend their free time, the majority (*n* = 317) engage in social media, followed by 256 who pursue hobbies, 199 who read books, 170 who focus on learning, 148 who participate in physical activity or sports, 138 who engage in gaming, 133 who spend time cooking, and 101 individuals who dedicate their free time to watching TV.

### 2.3. Measures

The Bergen Facebook Addiction Scale (BFAS) was adapted (the word “Facebook” was replaced with “TikTok”) to assess problematic TikTok use (PTTU) [79]. The scale includes six items that refer to six diagnostic criteria of addiction [80]: dominance, tolerance, mood change, relapse, withdrawal symptoms, and conflict (e.g., “Tried to cut down on the use of Facebook without success?”). Items are rated on a 5-point Likert scale (from 1 = very rarely to 5 = very often). The higher the score an individual achieves (maximum 30), the greater the TikTok addiction risk. Addiction can be considered to occur when at least three points are selected in all items. The BFAS scale was translated into several languages, including Polish [78,81,82], and research has demonstrated the excellent psychometric properties of the scale and its usefulness in assessing crucial diagnostic criteria of addiction [80]. The scale was previously modified by the authors of BFAS [26,78,83], and adapted versions were used in several other studies to assess addiction to various types of social media [6,68,84,85,86,87]. The adaptation (BSMAS) consisted of replacing the word “Facebook” in the instructions with a word denoting another social media (e.g., Instagram, Twitter, TikTok). The reliability coefficient in this study is Cronbach’s α = 0.86.

The Pure Procrastination Scale (PPS) was used to measure procrastination as a unidimensional construct [88,89]. The PPS includes 12 items (e.g., “I generally delay before starting on work I have to do”) with responses measured on a 5-point Likert scale, from 1 (describes me completely incorrectly) to 5 (describes me completely accurately). An international study (also conducted in Poland) has shown that the PPS is an effective tool to assess procrastination [90]. A high score (maximum 60 points) indicates a high level of procrastination (Cronbach’s α = 0.89 in the present study).

The 9-item Patient Health Questionnaire (PHQ-9) was used to assess the self-reported severity of depressive symptoms over the past two weeks [91,92]. The questionnaire covers a range of depressive symptoms, including low mood, loss of interest or pleasure, changes in sleep patterns, fatigue, changes in appetite or weight, feelings of guilt or worthlessness, difficulty concentrating, psychomotor agitation or retardation, and thoughts of death or suicide ideation (e.g., “Feeling bad about yourself–or that you are a failure or have let yourself or your family down”). The response options typically range from “0” (not at all) to “3” (nearly every day). Scores usually range from 0 to 27, with higher scores indicating more severe depressive symptoms, interpreted as 0–4 = minimal depression, 5–9 = mild depression, 10–14 = moderate depression, 15–19 = moderately severe depression, 20–27 = severe depression [91]. The reliability coefficient in this study is Cronbach’s α = 0.86. 

Demographic questions concerned gender (Men, Women), age (years), relationship status (Single, In a relationship), place of residence (Village, City up to 50 thousand people, City from 50 thousand up to 150 thousand people, City from 150 thousand up to 300 thousand people, City with over 300 thousand inhabitants), education (Primary, Vocational, Secondary, Higher), and professional status (I am a student, I study and work, Employed, Unemployed). There were also questions strictly related to the TikTok application: the amount of time spent using the TikTok application (Up to 1 h, 1–2 h, 3–4 h, and 5 h or more), the frequency of using TikTok (1/month, 1/week, 1/day, 5 times/day, 6–10 times/day, 10 times/day or more), and motives for visiting this application (Boredom, Entertainment, Curiosity, Needs to be up to date, To escape from their duties, problems or thoughts). The last question was about the forms of spending free time by the respondents.

### 2.4. Statistical Analysis

The descriptive analysis was performed for the total sample (N = 448), including a percentage of particular options in categorical responses to demographic questions and a range of scores, mean (M), standard deviation (SD), skewness, and kurtosis for continuous variables (procrastination, PTTU, and depression symptoms). The data met the criteria for parametric tests since the sample size was quite large (N > 200), and skewness and kurtosis ranged between +1 and −1 for all continuous variables (including procrastination, PTTU, and depression symptoms). The independent samples *t*-test was performed to examine gender differences in procrastination, PTTU, and depression severity. A Pearson’s correlation analysis examined associations between all scales of procrastination, PTTU, and depression severity. Structural equation modeling (SEM) was performed to examine the mediation model, with delta method standard errors and maximum likelihood (ML) estimator. A bias-corrected percentile bootstrap technique was applied, with 5000 sample replications and a 95% confidence interval, to increase the accuracy of the measurement. All statistical tests were performed using the JASP ver. 0.16.1.0. software for Windows.

## 3. Results

### 3.1. Gender and Age Differences in Procrastination, Problematic TikTok Use, and Depression Symptoms

As a sensitive analysis, the independent samples *t*-test was performed to examine gender and age differences in young adults. Statistically significant differences between men and women were not found in procrastination, PTTU, and depression symptoms severity (Table 2). Emerging adults (18–25 years old) scored significantly higher in depression symptoms than young adults (25–35 years old). No more significant differences were observed across the ages (Table 3).

### 3.2. Associations between Procrastination, Problematic TikTok Use, and Depression Symptoms

**Figure 1:**
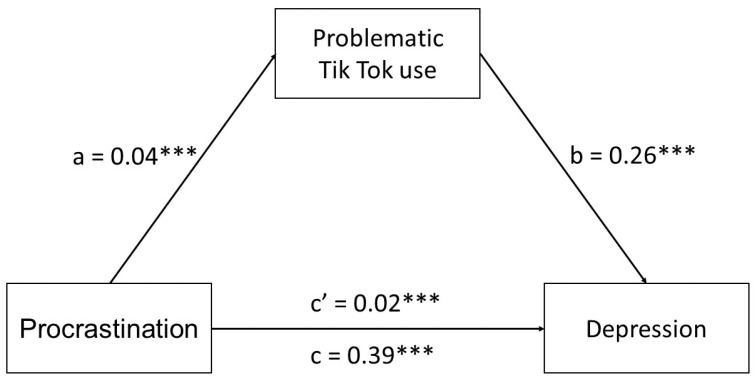
Indirect effect of procrastination on depression through problematic TikTok use. Standardized regression coefficients (β) are presented on the image. N = 448. *** *p* < 0.001.

Initially, we performed Pearson’s correlation analysis to examine the associations between procrastination, problematic TikTok use (PTTU), and depression symptoms. Procrastination was positively related to PTTU (*r* = 0.42, *p* < 0.001) and depression (*r* = 0.39, *p* < 0.001). Also, PTTU correlated positively with depression (*r* = 0.38, *p* < 0.001). Next, we verified the mediating role of PTTU on the relationship between procrastination and depression symptoms among emerging adults (Table 4 and Figure 1). The total, indirect, and direct effects were significant (Table 2), suggesting that PTTU partially mediates the relationships between procrastination and depression symptom severity.

## 4. Discussion

The study confirmed the direct effect of procrastination on depression symptoms and also the indirect effect through PTTU. The study extended the current literature by showing that the mechanism explaining the severity of depression symptoms among young adults is based on the influence of an individual predisposition to procrastination (considered a personality dimension) on the overuse of TikTok, which ultimately increases the symptoms of depression. The results of these studies confirm the theoretical assumptions of the I-PACE model, explaining the addictive behavior related to specific social media use [62]. Moreover, the results of the present research are in line with the previous observations about particular associations between procrastination, problematic social media use, and depression. 

### 4.1. Procrastination and Depression

Although procrastination is not a formal symptom of depression, it is a behavior often experienced by people living with depression [46,53,54,55,56]. Research has shown that procrastination is associated with various mental health challenges, including depression, anxiety, stress, and low self-esteem [44,45,46,47,48,53,54]. Also, academic procrastination was related to diminished academic performance and depression symptom severity in previous studies [93]. Procrastination is a prevalent issue that can have significant negative effects on an individual’s mental health, productivity, and overall well-being [45]. Key aspects of procrastination include the irrational tendency to delay tasks and self-regulation issues. Chronic procrastinators often experience problems finishing tasks, while situational procrastinators delay based on the task itself [51,52]. Therefore, procrastination can be considered as a trait [46]. Among the Big Five personality traits, conscientiousness may contribute to procrastinating behavior [46].

While procrastination can be a result of depression, it is not always clear which condition comes first, leading to a complex relationship between the two. When individuals experience low energy, lack of motivation, and difficulty concentrating, which are common symptoms of depression, they may be more prone to procrastination. However, the relationship between procrastination and depression can be complex with bidirectional cognitive and biological factors, with each potentially exacerbating the other. Procrastination can be a maladaptive coping mechanism for dealing with negative emotions, stress, or low mood. Individuals may delay tasks as a way to avoid facing emotional challenges, and this avoidance can contribute to feelings of guilt, inadequacy, and increased stress, common elements of depression. Chronic procrastination can lead to a cycle of reduced productivity and accomplishment as tasks pile up and remain incomplete. This sense of unfulfillment and failure to meet one’s goals can contribute to feelings of hopelessness and sadness, which are characteristic of depression. Individuals who consistently delay tasks may perceive themselves as incapable or inefficient, leading to a decline in self-esteem, which is a component of depression. Procrastination can also involve cognitive distortions, such as irrational thoughts about the self, tasks, or the future. These distortions align with cognitive patterns observed in depression and may contribute to the development or exacerbation of depressive symptoms. Some studies suggest that there may be shared neurobiological factors between procrastination and depression [94,95]. Both involve aspects of executive function and may be linked to neurotransmitter imbalances.

### 4.2. Procrastination, Problematic TikTok Use, and Depression

The association between TikTok usage and mental health problems is a topic of growing concern. There are several dispositional, sociocultural, and behavioral factors affecting social network site addiction [31,32,34,62,78]. People who use social media excessively feel compelled to check social media, spend a lot of time on social media sites, and simultaneously spend less time doing offline activities. They have mood swings, and predominantly negative emotions appear when they do not browse social media. It can lead to physical and psychological addiction by triggering the brain’s reward system to release dopamine, the “feel-good” chemical [23,60,62]. Similar to substance use disorder, symptoms of addictive social media use include mood modification, salience, tolerance, withdrawal symptoms, conflict, and relapse [31,32,78]. A flow experience was found to be a key risk factor for problematic TikTok use among adolescents [96].

The main motives for TikTok use are social interaction, self-expression (especially socially rewarding self-presentation), needs affordance, trendiness, escapist addiction, novelty, and archiving [70,97,98]. Cao et al. [99] proposed that the extent of social media addiction is influenced by individuals’ emotional and functional attachment to the platform. However, similar to other social media platforms, the excessive or uncontrollable usage of the TikTok app can lead to negative consequences in various areas of life, encountering harmful content and its impact on mental health. 

Research has shown that problematic TikTok use is associated with depression, social anxiety, boredom proneness, and distress intolerance [73,74,75]. A study involving Chinese high school students found that problematic TikTok use is positively linked to memory loss, depression, anxiety, and stress [71]. Also, a recent study indicates that Internet addiction (including TikTok use) is a positive predictor of depressive symptoms in high school students [72]. However, several longitudinal and experimental research indicated the reverse causal effect of depression on excessive social media use [100]. Some studies [33,41] suggest a bidirectional association between problematic social media use (PSMU) and mental health risk (stress, depression, anxiety, and loneliness), which is also consistent with the I-PACE model [62]. In particular, the longer the time spent on SMU, the worse mental health outcomes [41]. However, another longitudinal study [101] indicated that although an elevated level of PSMU substantially heightened depression severity, the level of depression did not have a significant impact on the PSMU. Similarly, Coyne et al. [69] found that early problematic cell phone use predicted later depression (but not the opposite) in adolescents and emerging adults. 

Several things explain the association between problematic TikTok use and depression. The addictive design of TikTok, which encourages users to spend significant amounts of time scrolling through content, can lead to excessive screen time. The studies suggest that flow experience has significant direct and indirect effects on TikTok and other social media addiction behavior [21,96]. Studies evidenced that enjoyment was positively associated with concentration and, in turn, with time distortion, leading to problematic TikTok use among adolescents [96]. The other possible way to link TikTok with depression is fear of missing out, which was found to be a key factor contributing to increased symptoms of anxiety and depression [102]. Some content on TikTok may involve sensitive topics, including mental health struggles or challenges. Constant exposure to such content may influence mood and contribute to feelings of sadness or hopelessness. In addition, the TikTok app can spread misinformation, which can lead to the development of unhealthy beliefs among users [21]. TikTok, like other social media platforms, can foster social comparison. Users may compare their lives, appearances, and achievements to those presented on the platform, leading to feelings of inadequacy or low self-esteem, factors associated with depression. Furthermore, TikTok often features trending challenges, events, or activities that users might fear missing out on. Using TikTok, especially late at night, can interfere with sleep patterns through bedtime procrastination [103]. Bedtime procrastination plays a mediating role in the association between smartphone addiction and depression among university students [103]. Sleep disturbances are associated with an increased risk of depression [104]. Social media platforms, including TikTok, can also be venues for cyberbullying. Negative comments, criticism, or online harassment experienced on TikTok may contribute to increased stress and depressive symptoms. 

Excessive social media use, in general, leads to increasing procrastination levels [57,58,61,64,65] and is linked to feelings of loneliness, anxiety, and depression [71,73,75]. The widespread presence and accessibility of temptations, such as TikTok use, can be seen as an occasion to put off essential matters until later and further intensify the issue of procrastination [46]. Individuals who engage with the TikTok platform may experience delays or postponements in completing tasks and responsibilities due to the addictive and time-consuming nature of the app. The captivating content on TikTok, in the form of short videos, can lead users to spend extended periods on the platform, potentially diverting their attention from more pressing or important activities. This association highlights the impact of social media platforms like TikTok on time management and productivity, contributing to procrastination tendencies among users. Indeed, the research indicated that university students who frequently procrastinated were more likely to exhibit problematic patterns of new media use and experience elevated levels of anxiety about the future [57]. Moreover, research indicated that passively measured smartphone use was related positively to procrastination among undergraduates [58]. However, individual differences were found related to specific application categories (social media, messaging, browsers, gaming, and video streaming). Using the technology habit approach, Meier [65] found that mobile checking habits increase procrastination (small to moderate correlations), which, in turn, decreases affective well-being. However, the association between mobile habits and the effect on well-being was minimal. 

On the other hand, procrastination was previously considered a causal factor of PSMU in longitudinal studies [63,105]. According to a survey, 67% of students said that TikTok caused them to procrastinate (e.g., not do homework for school), and 69% of students said they felt addicted to the app [59]. TikTok can cause users to lose focus and not complete their tasks, leading to poor academic performance. The app’s algorithm, which sends videos to a set number of people based on user interactions, can make it difficult for users to stop consuming content, contributing to procrastination [59]. The mediating role of general procrastination behaviors in the relationship between self-control and social media addiction was confirmed previously in a large sample of Turkish university students [106]. Davis [7] emphasized in the Cognitive-Behavioral Model of Pathological Internet Use (CBMPIU) that procrastination plays a pivotal role in the social media addiction process. Excessive internet users tend to defer responsibilities, leading to increased pressure and challenges in daily functioning. Indeed, recent research confirmed that the unpleasant state of withdrawal forms the foundation for the utilization of short-form videos as a means to evade tasks [60]. Consequently, procrastination holds the potential to elevate the susceptibility to addiction. 

### 4.3. The Role of Gender and Age

The present study found no significant gender differences in procrastination, PTTU, and depression symptoms. Younger participants (between 18 and 25 years of age) showed higher depression levels than older samples (ranging in age between 26 and 35 years). The current results are partially in line with previous studies. A large national study in Germany [47] showed that significantly higher procrastination levels are demonstrated in younger (aged 14–29) rather than older people and males rather than females (but only in the youngest group, up to 30 years of age). However, no gender differences in procrastination were found among adults over 30 years old. It is possible that some intergenerational differences (in a German study conducted in 2016) impacted the current discrepancy. However, no significant gender differences in self-reported chronic procrastination dimensions (arousal and avoidant procrastination) were found in previous international studies [52], which is consistent with our study.

The study did not find gender differences in TikTok use. Although gender differences in social media use (SMU) were found in a systematic review, they were related to the specific SMU [41]. For example, the addictive use of video games was more prevalent in men, whereas women most frequently presented an addictive use of social media in a large sample of 23,533 adults [26]. Since TikTok use was not examined across different genders, we had no specific hypothesis about this issue. In addition, a recent meta-analysis did not find evidence of heterogeneity of correlations between problematic social media use and adverse mental health outcomes by age, gender, or year of publication [36]. Therefore, the present study seems to be consistent with previous research to some extent.

However, the lack of gender differences in depression is inconsistent with a large body of previous studies. The WHO’s [2] global study found significant gender differences, with women scoring higher than men for depression symptoms. This discrepancy can be a result of cross-cultural differences. On the other hand, only those people who used TikTok participated in the study. It is important to note that not everyone who uses TikTok and procrastinates will experience depression. Conversely, some TikTok users may find the platform entertaining and enjoyable without a significant impact on their mental health. Moderation, self-awareness, and a balanced approach to social media use are advisable to promote mental well-being. More studies are required to explain the current results.

The study found a higher severity of depression symptoms in emerging adults compared to those young adults whose ages ranged between 26 and 35 years, but the effect size was small. This result is consistent with previous studies. Kuwabara et al. [4] showed the highest incidence and cumulative prevalence of depression among emerging adults than older adults. The rise in depression and anxiety among young adults is a complicated issue with major contributing factors, including more significant levels of social media engagement, academic stress, and economic stress [5,107,108]. Emerging adults may face unique challenges and stressors as they navigate the transition from adolescence to full adulthood [109]. Depression during this period can manifest in various ways, impacting emotional well-being, relationships, and overall functioning [110]. Unfortunately, depression during the early stages of adulthood affects subsequent labor market outcomes by diminishing work experience and shaping the choice of occupations marked by skill distributions associated with lower potential for wage growth [111]. Therefore, recognizing the distinct challenges and risk factors of depression in young adults is a crucial problem to resolve.

### 4.4. Practical Implications

The mechanism of interplay between procrastination, PTTU, and depression can be helpful in preparation for prevention and intervention programs among young adults. Addressing problematic TikTok use and procrastination and seeking support for mental health concerns, such as depression, can be important steps in improving the overall well-being of young adults. Meta-analysis also indicated that psychological interventions are adequate for the prevention and treatment of depression and other mental problems (including procrastination and internet use) in college students [112]. In particular, procrastination can be an essential part of managing depression and other mental health conditions. Cognitive-behavioral therapy was found to be an effective treatment for people struggling with severe procrastination [113]. Perversely, the Internet and mobile phones can be used to reduce mental problems. A systematic review [114] showed that mobile phone apps can reduce problematic smartphone usage. Depression severity decreased on average by 20% when a self-guided computerized cognitive behavioral tool was used during COVID-19 [115]. The study suggests that Internet- and mobile-based interventions are an effective treatment, reducing symptoms of internet addiction as well as depressive symptoms, anxiety, and procrastination behavior [116,117].

### 4.5. Study Limitations

Although the present study improved the knowledge about the relationships between procrastination, PTTU, and depression, the cross-sectional nature of this study prevents causal solid relationships. Longitudinal research should be performed to confirm the mediation model fully. Although a large sample size was included in the study, the online survey in one country may not be fully representative of all TikTok users. The sample included young adults aged 18–35, predominantly university students; therefore, the results cannot be generalized to adolescents, middle-aged, and older people. More international research is required to explore cross-cultural, gender, and age-related potential differences (adolescents, middle-aged, and older adults) in procrastination, TikTok use, depression, and its associations. It is crucial to recognize that these associations are complex, and individual experiences can vary. Additionally, various factors, including individual differences, life circumstances, and coping mechanisms, can influence the nature and strength of this association. More complex studies could be performed in the future to control more variables as potential factors of depression, especially among young people. Although the indirect effect in the mediation model was significant regarding *p*-value and bias-corrected bootstrap confidence interval, considering the practical interpretation of effect size estimates for the clinical practice in social sciences, only the total effect of procrastination on depression and direct effect of PTTU on depression showed recommended minimal significance [118]. The relationship between procrastination and PTTU can be interpreted as insignificant for clinical practice. Therefore, the present results should be considered with caution until the other research confirms these effects with a stronger justification in effect size.

## 5. Conclusions

The study suggests that a tendency to procrastinate may lead to increased use of TikTok and, subsequently, heightened symptoms of depression. No gender differences were identified regarding procrastination, problematic TikTok use, and depression. Emerging adults experienced more severe depression symptoms than their counterparts aged 29–35 years old, but both groups showed similar levels of procrastination and problematic TikTok use. This study contributes to a deeper understanding of the relationship between procrastination and problematic social media use and their impact on mental health. Our findings support the I-PACE model of addictive behavior related to social media use. To alleviate depression symptoms among young adults, prevention and intervention programs should focus on addressing both procrastination and excessive TikTok use. The complex treatment of procrastination, TikTok use, and depression could be most effective in improving the well-being of young adults.

## Figures and Tables

**Table 1:** Participant characteristics.

Variable	Categories of Variable	*n*	%
Gender	Men	214	47.77
	Women	234	52.23
Relationship status	In relationship	248	55.36
	Single	200	44.64
Education	Primary	9	2.01
	Vocational	9	2.01
	Secondary	231	51.56
	Higher	199	44.42
Place of residence	Village	82	18.30
	City up to 50 thousand people	75	16.74
	City from 50 thousand up to 150 thousand of people	71	15.85
	City from 150 thousand up to 300 thousand of people	97	21.65
	A city with over 300 thousand inhabitants	123	27.46
Professional status	I am a student	231	51.56
	I study and work	95	21.21
	Employed	112	25.00
	Unemployed	10	2.23
Time to spend on using TikTok a day	Up to one hour	172	38.39
	Between one and two hours	138	30.80
	Between three and four hours	112	25.00
	Five hours or more	26	5.80
Frequency of using TikTok	Once a month	51	11.38
	Once a week	41	9.15
	Once a day	93	20.76
	Five times a day	156	34.82
	From six up to ten times a day	71	15.85
	More than ten times a day	36	8.04
Motives to use TikTok	Boredom	133	29.69
	Entertainment	147	32.81
	Curiosity	58	12.95
	Need to be up to date	27	6.03
	To escape from their duties, problems, or thoughts	83	18.53

**Table 2:** Gender differences in mean scores of procrastination, problematic TikTok use, and depression symptoms.

	Men (*n* = 214)		Women (*n* = 234)				
Variables	*M*	*SD*	*M*	*SD*	*t*(446)	*p*	*d*
Problematic TikTok use	13.99	6.06	14.77	5.47	–1.45	0.149	–0.137
Procrastination	32.98	11.09	32.88	11.34	0.10	0.921	0.009
Depression	8.95	5.73	9.48	5.81	–0.97	0.332	–0.092

**Table 3:** Differences between young and emerging adults in procrastination, problematic TikTok use, and depression symptoms.

	Young Adults (Age > 25, *n* = 138)		Emerging Adults (Age ≤ 25, *n* = 310)				
Variables	*M*	*SD*	*M*	*SD*	*t*(446)	*p*	*d*
Problematic TikTok use	14.72	5.41	14.26	5.92	0.78	0.434	0.080
Procrastination	32.17	10.63	33.26	11.46	–0.95	0.344	–0.097
Depression	8.12	5.03	9.72	6.01	–2.74	0.006	–0.280

**Table 4:** Parameter estimates for the association between procrastination, problematic TikTok use (PTTU), and depression in emerging adults (*N* = 448).

Effect	Predictor	Mediator	Outcome	β	*SE* β	*b*	*SE b*	*z*-Value	*p*	Bc_p_ 95% CI	
										LL	UL
	PTTU		Depression	0.26	0.05	0.26	0.05	5.69	<0.001	0.16	0.37
	Procrastination		Depression	0.03	0.00	0.14	0.02	6.01	<0.001	0.09	0.20
	Procrastination		PTTU	0.04	0.00	0.22	0.02	9.84	<0.001	0.17	0.26
Total	Procrastination		Depression	0.04	0.00	0.20	0.02	8.95	<0.001	0.15	0.25
Indirect	Procrastination	PTTU	Depression	0.01	0.00	0.06	0.01	4.92	<0.001	0.03	0.09
Direct	Procrastination		Depression	0.03	0.00	0.14	0.02	6.01	<0.001	0.09	0.20

Note: PTTU = problematic TikTok use, Bc_p_ = bias-corrected percentile bootstrap, CI = confidence intervals, LL = lower levels, UL = upper levels.

## Data Availability

The data presented in this study are available on request from the corresponding author.

## References

[1] Bettmann J.E., Anstadt G., Casselman B., Ganesh K. (2021). Young Adult Depression and Anxiety Linked to Social Media Use: Assessment and Treatment. Clin. Soc. Work J..

[2] World Health Organization Depression Fact Sheet 2023. https://www.who.int/news-room/fact-sheets/detail/depression.

[3] Goodwin R.D., Dierker L.C., Wu M., Galea S., Hoven C.W., Weinberger A.H. (2022). Trends in U.S. Depression Prevalence From 2015 to 2020: The Widening Treatment Gap. Am. J. Prev. Med..

[4] Kuwabara S.A., Van Voorhees B.W., Gollan J.K., Alexander G.C. (2007). A Qualitative Exploration of Depression in Emerging Adulthood: Disorder, Development, and Social Context. Gen. Hosp. Psychiatry.

[5] Berry D. (2004). The Relationship between Depression and Emerging Adulthood: Theory Generation. ANS Adv. Nurs. Sci..

[6] Bányai F., Zsila Á., Király O., Maraz A., Elekes Z., Griffiths M.D., Andreassen C.S., Demetrovics Z. (2017). Problematic Social Media Use: Results from a Large-Scale Nationally Representative Adolescent Sample. PLoS ONE.

[7] Davis R.A. (2001). Cognitive-Behavioral Model of Pathological Internet Use. Comput. Hum. Behav..

[8] Davis R.A., Flett G.L., Besser A. (2002). Validation of a New Scale for Measuring Problematic Internet Use: Implications for Pre-Employment Screening. Cyberpsychol. Behav..

[9] Boer M., van den Eijnden R.J.J.M., Boniel-Nissim M., Wong S.L., Inchley J.C., Badura P., Craig W.M., Gobina I., Kleszczewska D., Klanšček H.J. (2020). Adolescents’ Intense and Problematic Social Media Use and Their Well-Being in 29 Countries. J. Adolesc. Health.

[10] Brailovskaia J., Rohmann E., Bierhoff H.W., Margraf J., Köllner V. (2019). Relationships between Addictive Facebook Use, Depressiveness, Insomnia, and Positive Mental Health in an Inpatient Sample: A German Longitudinal Study. J. Behav. Addict..

[11] Brailovskaia J., Margraf J. (2020). Relationship between Depression Symptoms, Physical Activity, and Addictive Social Media Use. Cyberpsychol. Behav. Soc. Netw..

[12] Marino C., Gini G., Vieno A., Spada M.M. (2018). The Associations between Problematic Facebook Use, Psychological Distress and Well-Being among Adolescents and Young Adults: A Systematic Review and Meta-Analysis. J. Affect. Disord..

[13] Ryding F.C., Kuss D.J. (2020). Passive Objective Measures in the Assessment of Problematic Smartphone Use: A Systematic Review. Addict. Behav. Rep..

[14] Carr C.T., Hayes R.A. (2015). Social Media: Defining, Developing, and Divining. Atl. J. Commun..

[15] Obar J.A., Wildman S. (2015). Social Media Definition and the Governance Challenge: An Introduction to the Special Issue. Telecommun. Policy.

[16] Karakose T., Yıldırım B., Tülübaş T., Kardas A. (2023). A Comprehensive Review on Emerging Trends in the Dynamic Evolution of Digital Addiction and Depression. Front. Psychol..

[17] Vogels E.A., Gelles-Watnick R., Massarat N. (2022). Teens, Social Media and Technology 2022. https://policycommons.net/artifacts/2644169/teens-social-media-and-technology-2022/3667002/.

[18] Liang X. Research on How to Perceive Their Behavior for International High School Students Based on Using TikTok with Semi-Structured Interview. Proceedings of the 2021 6th International Conference on Social Sciences and Economic Development (ICSSED 2021).

[19] Xu L., Yan X., Zhang Z. (2019). Research on the Causes of the “Tik Tok” App Becoming Popular and the Existing Problems. J. Adv. Manag. Sci..

[20] Montag C., Yang H., Elhai J.D. (2021). On the Psychology of TikTok Use: A First Glimpse From Empirical Findings. Front. Public Health.

[21] Qin Y., Omar B., Musetti A. (2022). The Addiction Behavior of Short-Form Video App TikTok: The Information Quality and System Quality Perspective. Front. Psychol..

[22] Zhao Z. (2021). Analysis on the Douyin (Tiktok) Mania Phenomenon Based on Recommendation Algorithms. E3S Web Conf..

[23] Pedrouzo S.B., Krynski L. (2023). Hyperconnected: Children and Adolescents on Social Media. The TikTok Phenomenon. Arch. Argent. Pediatr..

[24] Bucknell Bossen C., Kottasz R. (2020). Uses and Gratifications Sought by Pre-Adolescent and Adolescent TikTok Consumers. Young Consum..

[25] Yang Z., Griffiths M.D., Yan Z., Xu W. (2021). Can Watching Online Videos Be Addictive? A Qualitative Exploration of Online Video Watching among Chinese Young Adults. Int. J. Envrion. Res. Public Health.

[26] Andreassen C.S., Billieux J., Griffiths M.D., Kuss D.J., Demetrovics Z., Mazzoni E., Pallesen S. (2016). The Relationship between Addictive Use of Social Media and Video Games and Symptoms of Psychiatric Disorders: A Large-Scale Cross-Sectional Study. Psychol. Addict. Behav..

[27] Busch P.A., McCarthy S. (2021). Antecedents and Consequences of Problematic Smartphone Use: A Systematic Literature Review of an Emerging Research Area. Comput. Hum. Behav..

[28] McLachlan S. The 50+ Important TikTok Stats Marketers Need to Know. https://blog.hootsuite.com/tiktok-stats/.

[29] Petrović Z.K., Peraica T., Kozarić-Kovačić D., Palavra I.R. (2022). Internet Use and Internet-Based Addictive Behaviours during Coronavirus Pandemic. Curr. Opin. Psychiatry.

[30] Haddad J.M., Macenski C., Mosier-Mills A., Hibara A., Kester K., Schneider M., Conrad R.C., Liu C.H. (2021). The Impact of Social Media on College Mental Health During the COVID-19 Pandemic: A Multinational Review of the Existing Literature. Curr. Psychiatry Rep..

[31] Kuss D.J., Griffiths M.D. (2011). Online Social Networking and Addiction-A Review of the Psychological Literature. Int. J. Envrion. Res. Public Health.

[32] Kuss D.J., Griffiths M.D. (2017). Social Networking Sites and Addiction: Ten Lessons Learned. Int. J. Envrion. Res. Public Health.

[33] Zhou W., Yan Z., Yang Z., Hussain Z. (2023). Problematic Social Media Use and Mental Health Risks among First-Year Chinese Undergraduates: A Three-Wave Longitudinal Study. Front. Psychiatry.

[34] Rathod A.S., Ingole A., Gaidhane A., Choudhari S.G. (2022). Psychological Morbidities Associated With Excessive Usage of Smartphones Among Adolescents and Young Adults: A Review. Cureus.

[35] Huang C. (2022). A Meta-Analysis of the Problematic Social Media Use and Mental Health. Int. J. Soc. Psychiatry.

[36] Shannon H., Bush K., Villeneuve P.J., Hellemans K.G., Guimond S. (2022). Problematic Social Media Use in Adolescents and Young Adults: Systematic Review and Meta-Analysis. JMIR Ment. Health.

[37] Yoon S., Kleinman M., Mertz J., Brannick M. (2019). Is Social Network Site Usage Related to Depression? A Meta-Analysis of Facebook–Depression Relations. J. Affect. Disord..

[38] Hussain Z., Wegmann E., Yang H., Montag C. (2020). Social Networks Use Disorder and Associations With Depression and Anxiety Symptoms: A Systematic Review of Recent Research in China. Front. Psychol..

[39] Elhai J.D., Dvorak R.D., Levine J.C., Hall B.J. (2017). Problematic Smartphone Use: A Conceptual Overview and Systematic Review of Relations with Anxiety and Depression Psychopathology. J. Affect. Disord..

[40] Keles B., McCrae N., Grealish A. (2020). A Systematic Review: The Influence of Social Media on Depression, Anxiety and Psychological Distress in Adolescents. Int. J. Adolesc. Youth.

[41] Lopes L.S., Valentini J.P., Monteiro T.H., De Freitas Costacurta M.C., Soares L.O.N., Telfar-Barnard L., Nunes P.V. (2022). Problematic Social Media Use and Its Relationship with Depression or Anxiety: A Systematic Review. Cyberpsychol. Behav. Soc. Netw..

[42] Bekalu M.A., Sato T., Viswanath K. (2023). Conceptualizing and Measuring Social Media Use in Health and Well-Being Studies: Systematic Review. J. Med. Internet Res..

[43] Huang C. (2017). Time Spent on Social Network Sites and Psychological Well-Being: A Meta-Analysis. Cyberpsychol. Behav. Soc. Netw..

[44] Sirois F.M., Pychyl T.A. (2016). Procrastination. Encyclopedia of Mental Health.

[45] Ferrari J.R., Tibbett T.P. (2020). Procrastination. Encyclopedia of Personality and Individual Differences.

[46] Steel P. (2007). The Nature of Procrastination: A Meta-Analytic and Theoretical Review of Quintessential Self-Regulatory Failure. Psychol Bull..

[47] Beutel M.E., Klein E.M., Aufenanger S., Brähler E., Dreier M., Müller K.W., Quiring O., Reinecke L., Schmutzer G., Stark B. (2016). Procrastination, Distress and Life Satisfaction across the Age Range—A German Representative Community Study. PLoS ONE.

[48] Ma X., Li Z., Lu F. (2023). The Influence of Stressful Life Events on Procrastination among College Students: Multiple Mediating Roles of Stress Beliefs and Core Self-Evaluations. Front. Psychol..

[49] Yan B., Zhang X. (2022). What Research Has Been Conducted on Procrastination? Evidence From a Systematical Bibliometric Analysis. Front. Psychol..

[50] Kim K.R., Seo E.H. (2015). The Relationship between Procrastination and Academic Performance: A Meta-Analysis. Pers. Individ. Differ..

[51] Steel P., Ferrari J. (2013). Sex, Education and Procrastination: An Epidemiological Study of Procrastinators’ Characteristics from a Global Sample. Eur. J. Pers..

[52] Ferrari J.R., Díaz-Morales J.F., O’Callaghan J., Díaz K., Argumedo D. (2007). Frequent Behavioral Delay Tendencies by Adults: International Prevalence Rates of Chronic Procrastination. J. Cross Cult. Psychol..

[53] Balkis M., Duru E. (2017). Gender Differences in the Relationship between Academic Procrastination, Satifaction with Academic Life and Academic Performance. Electron. J. Res. Educ. Psychol..

[54] Duru E., Balkis M. (2017). Procrastination, Self-Esteem, Academic Performance, and Well-Being: A Moderated Mediation Model. Int. J. Educ. Psychol..

[55] Uzun Ozer B., O’Callaghan J., Bokszczanin A., Ederer E., Essau C. (2014). Dynamic Interplay of Depression, Perfectionism and Self-Regulation on Procrastination. Br. J. Guid. Counc..

[56] Cjuno J., Palomino-Ccasa J., Silva-Fernandez R.G., Soncco-Aquino M., Lumba-Bautista O., Hernández R.M. (2023). Academic Procrastination, Depressive Symptoms and Suicidal Ideation in University Students: A Look during the Pandemic. Iran. J. Psychiatry.

[57] Przepiorka A., Blachnio A., Cudo A. (2023). Procrastination and Problematic New Media Use: The Mediating Role of Future Anxiety. Curr. Psychol..

[58] Aalbers G., vanden Abeele M.M.P., Hendrickson A.T., de Marez L., Keijsers L. (2022). Caught in the Moment: Are There Person-Specific Associations between Momentary Procrastination and Passively Measured Smartphone Use?. Mob. Media Commun..

[59] Holzmann Z. (2021). The Ugly Side of TikTok. *Cardinal*. https://wearecardinals.com/1584/showcase/the-ugly-side-of-tiktok/.

[60] Tian X., Bi X., Chen H. (2023). How Short-Form Video Features Influence Addiction Behavior? Empirical Research from the Opponent Process Theory Perspective. Inf. Technol. People.

[61] Rozgonjuk D., Kattago M., Täht K. (2018). Social Media Use in Lectures Mediates the Relationship between Procrastination and Problematic Smartphone Use. Comput. Hum. Behav..

[62] Brand M., Young K.S., Laier C., Wölfling K., Potenza M.N. (2016). Integrating Psychological and Neurobiological Considerations Regarding the Development and Maintenance of Specific Internet-Use Disorders: An Interaction of Person-Affect-Cognition-Execution (I-PACE) Model. Neurosci. Biobehav. Rev..

[63] Lardinoix J., Neumann I., Wartberg L., Lindenberg K. (2023). Procrastination Predicts Future Internet Use Disorders in Adolescents but Not Vice Versa: Results from a 12-Month Longitudinal Study. Healthcare.

[64] Meier A., Reinecke L., Meltzer C.E. (2016). “Facebocrastination”? Predictors of Using Facebook for Procrastination and Its Effects on Students’ Well-Being. Comput. Hum. Behav..

[65] Meier A. (2022). Studying Problems, Not Problematic Usage: Do Mobile Checking Habits Increase Procrastination and Decrease Well-Being?. Mob. Media Commun..

[66] Hinsch C., Sheldon K.M. (2013). The Impact of Frequent Social Internet Consumption: Increased Procrastination and Lower Life Satisfaction. J. Consum. Behav..

[67] Elhai J.D., Sapci O., Yang H., Amialchuk A., Rozgonjuk D., Montag C. (2021). Objectively-Measured and Self-Reported Smartphone Use in Relation to Surface Learning, Procrastination, Academic Productivity, and Psychopathology Symptoms in College Students. Hum. Behav. Emerg. Technol..

[68] Shensa A., Escobar-Viera C.G., Sidani J.E., Bowman N.D., Marshal M.P., Primack B.A. (2017). Problematic Social Media Use and Depressive Symptoms among U.S. Young Adults: A Nationally-Representative Study. Soc. Sci. Med..

[69] Coyne S.M., Stockdale L., Summers K. (2019). Problematic Cell Phone Use, Depression, Anxiety, and Self-Regulation: Evidence from a Three Year Longitudinal Study from Adolescence to Emerging Adulthood. Comput. Hum. Behav..

[70] Smith T., Short A. (2022). Needs Affordance as a Key Factor in Likelihood of Problematic Social Media Use: Validation, Latent Profile Analysis and Comparison of TikTok and Facebook Problematic Use Measures. Addict. Behav..

[71] Sha P., Dong X. (2021). Research on Adolescents Regarding the Indirect Effect of Depression, Anxiety, and Stress between Tiktok Use Disorder and Memory Loss. Int. J. Envrion. Res. Public Health.

[72] Ilić-živojinović J.B., Mitić T., Srećković M., Backović D., Soldatović I. (2023). The Relationship between Internet Use and Depressive Symptoms among High School Students. Srp. Arh. Celok. Lek..

[73] Günlü A., Oral T., Yoo S., Chung S. (2023). Reliability and Validity of the Problematic TikTok Use Scale among the General Population. Front. Psychiatry.

[74] Gupta A.K., Upreti D., Shrestha S., Sawant S., Karki U., Shoib S. (2021). Adolescent-Parent Conflict in the Era of ‘TikTok’: Case Reports from Nepal. J. Affect. Disord. Rep..

[75] Yao N., Chen J., Huang S., Montag C., Elhai J.D. (2023). Depression and Social Anxiety in Relation to Problematic TikTok Use Severity: The Mediating Role of Boredom Proneness and Distress Intolerance. Comput. Hum. Behav..

[76] Mu A., Yuan S., Liu Z. (2023). Internet Use and Depressive Symptoms among Chinese Older Adults: Two Sides of Internet Use. Front. Public Health.

[77] Pychyl T.A., Flett G.L. (2012). Procrastination and Self-Regulatory Failure: An Introduction to the Special Issue. J. Ration.-Emotive Cogn.-Behav. Ther..

[78] Andreassen C.S. (2015). Online Social Network Site Addiction: A Comprehensive Review. Curr. Addict. Rep..

[79] Andreassen C.S., TorbjØrn T., Brunborg G.S., Pallesen S. (2012). Development of a Facebook Addiction Scale. Psychol. Rep..

[80] Griffiths M. (2005). A ‘Components’ Model of Addiction within a Biopsychosocial Framework. J. Subst. Use.

[81] Atroszko P.A., Balcerowska J.M., Bereznowski P., Biernatowska A., Pallesen S., Schou Andreassen C. (2018). Facebook Addiction among Polish Undergraduate Students: Validity of Measurement and Relationship with Personality and Well-Being. Comput. Hum. Behav..

[82] Góźdź J., Charzyńska E. (2014). W Sieci Uzależnienia: Polska Adaptacja Skali Uzależnienia Od Facebooka (the Bergen Facebook Addiction Scale) C.S. Andreassen, T. Torsheima, G.S. Brunborga i S. Pallesena. Chowanna.

[83] Andreassen C.S., Pallesen S., Griffiths M.D. (2017). The Relationship between Addictive Use of Social Media, Narcissism, and Self-Esteem: Findings from a Large National Survey. Addict. Behav..

[84] Kircaburun K., Griffiths M.D. (2018). The Dark Side of Internet: Preliminary Evidence for the Associations of Dark Personality Traits with Specific Online Activities and Problematic Internet Use. J. Behav. Addict..

[85] Lee S.L. (2019). Predicting SNS Addiction with the Big Five and the Dark Triad. Cyberpsychol.-J. Psychosoc. Res. Cyberspace.

[86] Shensa A., Sidani J.E., Dew M.A., Escobar-Viera C.G., Primack B.A. (2018). Social Media Use and Depression and Anxiety Symptoms: A Cluster Analysis. Am. J. Health Behav..

[87] Rogowska A.M., Libera P. (2022). Life Satisfaction and Instagram Addiction among University Students during the COVID-19 Pandemic: The Bidirectional Mediating Role of Loneliness. Int. J. Envrion. Res. Public Health.

[88] Steel P. (2010). Arousal, Avoidant and Decisional Procrastinators: Do They Exist?. Pers. Individ. Differ..

[89] Stępień M., Topolewska E., Topolewska E., Skinina E., Skrzek S. (2014). Identity Styles According to Berzonsky and Procrastination. Young Psychology.

[90] Svartdal F., Pfuhl G., Nordby K., Foschi G., Klingsieck K.B., Rozental A., Carlbring P., Lindblom-Ylänne S., Rebkowska K. (2016). On the Measurement of Procrastination: Comparing Two Scales in Six European Countries. Front. Psychol..

[91] Kroenke K., Spitzer R.L., Williams J.B.W. (2001). The PHQ-9: Validity of a Brief Depression Severity Measure. J. Gen. Intern. Med..

[92] Kokoszka A., Jastrzębski A., Obrębski M. (2016). Ocena Psychometrycznych Właściwości Polskiej Wersji Kwestionariusza Zdrowia Pacjenta-9 Dla Osób Dorosłych. Psychiatria.

[93] Nazari S., Ajorpaz N., Sadat Z., Hosseinian M., Esalatmanesh S. (2021). The Relationship between Academic Procrastination and Depression in Students of Kashan University of Medical Sciences. Int. Arch. Health Sci..

[94] Gustavson D.E., Miyake A., Hewitt J.K., Friedman N.P. (2014). Genetic Relations among Procrastination, Impulsivity, and Goal-Management Ability: Implications for the Evolutionary Origin of Procrastination. Psychol. Sci..

[95] Gustavson D.E., du Pont A., Hatoum A.S., Rhee S.H., Kremen W.S., Hewitt J.K., Friedman N.P. (2017). Genetic and Environmental Associations Between Procrastination and Internalizing/Externalizing Psychopathology. Clin. Psychol. Sci..

[96] Qin Y., Musetti A., Omar B. (2023). Flow Experience Is a Key Factor in the Likelihood of Adolescents’ Problematic TikTok Use: The Moderating Role of Active Parental Mediation. Int. J. Environ. Res. Public Health.

[97] Omar B., Dequan W. (2020). Watch, Share or Create: The Influence of Personality Traits and User Motivation on TikTok Mobile Video Usage. Int. J. Interact. Mob. Technol..

[98] Gu L., Gao X., Li Y. (2022). What Drives Me to Use TikTok: A Latent Profile Analysis of Users’ Motives. Front. Psychol..

[99] Cao X., Gong M., Yu L., Dai B. (2020). Exploring the Mechanism of Social Media Addiction: An Empirical Study from WeChat Users. Internet Res..

[100] Hartanto A., Quek F.Y.X., Tng G.Y.Q., Yong J.C. (2021). Does Social Media Use Increase Depressive Symptoms? A Reverse Causation Perspective. Front. Psychiatry.

[101] Chang C.W., Huang R.Y., Strong C., Lin Y.C., Tsai M.C., Chen I.H., Lin C.Y., Pakpour A.H., Griffiths M.D. (2022). Reciprocal Relationships Between Problematic Social Media Use, Problematic Gaming, and Psychological Distress Among University Students: A 9-Month Longitudinal Study. Front. Public Health.

[102] Elhai J.D., Yang H., Fang J., Bai X., Hall B.J. (2020). Depression and Anxiety Symptoms Are Related to Problematic Smartphone Use Severity in Chinese Young Adults: Fear of Missing out as a Mediator. Addict. Behav..

[103] Geng Y., Gu J., Wang J., Zhang R. (2021). Smartphone Addiction and Depression, Anxiety: The Role of Bedtime Procrastination and Self-Control. J. Affect. Disord..

[104] Feng Y., Meng D., Guo J., Zhao Y., Ma X., Zhu L., Mu L. (2022). Bedtime Procrastination in the Relationship between Self-Control and Depressive Symptoms in Medical Students: From the Perspective of Sex Differences. Sleep Med..

[105] Hong W., Liu R.D., Ding Y., Jiang S., Yang X., Sheng X. (2021). Academic Procrastination Precedes Problematic Mobile Phone Use in Chinese Adolescents: A Longitudinal Mediation Model of Distraction Cognitions. Addict. Behav..

[106] Ekşi H., Turgut T., Sevim E. (2019). The Mediating Role of General Procrastination Behaviors in the Relationship between Self-Control and Social Media Addiction in University Students. Addicta Turk. J. Addict..

[107] Mondi C.F., Reynolds A.J., Ou S.R. (2017). Predictors of Depressive Symptoms in Emerging Adulthood in a Low-Income Urban Cohort. J. Appl. Dev. Psychol..

[108] Pilar Matud M., Díaz A., Bethencourt J.M., Ibáñez I. (2020). Stress and Psychological Distress in Emerging Adulthood: A Gender Analysis. J. Clin. Med..

[109] Arnett J.J. (2014). Emerging Adulthood: The Winding Road from the Late Teens Through the Twenties.

[110] Brito A.D., Soares A.B. (2023). Well-Being, Character Strengths, and Depression in Emerging Adults. Front. Psychol..

[111] Wang B., Frank R.G., Glied S.A., Wagner R.F. (2022). Lasting Scars: The Impact of Depression in Early Adulthood on Subsequent Labor Market Outcomes.

[112] Cuijpers P., Miguel C., Ciharova M., Aalten P., Batelaan N., Salemink E., Spinhoven P., Struijs S., de Wit L., Gentili C. (2021). Prevention and Treatment of Mental Health and Psychosocial Problems in College Students: An Umbrella Review of Meta-Analyses. Clin. Psychol. Sci. Pract..

[113] Rozental A., Forsström D., Lindner P., Nilsson S., Mårtensson L., Rizzo A., Andersson G., Carlbring P. (2018). Treating Procrastination Using Cognitive Behavior Therapy: A Pragmatic Randomized Controlled Trial Comparing Treatment Delivered via the Internet or in Groups. Behav. Ther..

[114] Bychkov D., Young S.D. (2018). Facing Up to Nomophobia: A Systematic Review of Mobile Phone Apps That Reduce Smartphone Usage. Stud. Big Data.

[115] Guarino I.D., Cowan D.R., Fellows A.M., Buckey J.C. (2021). Use of a Self-Guided Computerized Cognitive Behavioral Tool During COVID-19: Evaluation Study. JMIR Form. Res..

[116] Bernstein K., Zarski A.C., Pekarek E., Schaub M.P., Berking M., Baumeister H., Ebert D.D. (2023). Case Report for an Internet- and Mobile-Based Intervention for Internet Use Disorder. Front. Psychiatry.

[117] Miralles I., Granell C., Díaz-Sanahuja L., van Woensel W., Bretón-López J., Mira A., Castilla D., Casteleyn S. (2020). Smartphone Apps for the Treatment of Mental Disorders: Systematic Review. JMIR mHealth uHealth.

[118] Ferguson C.J. (2009). An Effect Size Primer: A Guide for Clinicians and Researchers. Prof. Psychol. Res. Pract..

